# Person, Population, Mechanism. Three Main Branches of Psychological Science

**DOI:** 10.17505/jpor.2023.25814

**Published:** 2023-12-07

**Authors:** Lars-Gunnar Lundh

**Affiliations:** Department of Psychology, Lund University

**Keywords:** persons, populations, mechanisms, psychological science, meta-perspective

## Abstract

There are different ways of dividing psychology into subdisciplines. The purpose of the present paper is to explore one specific meta-perspective on psychological science, seen as having three main branches: *person* psychology, *population* psychology, and *mechanism* psychology, linked to three different levels of research. Person-level research focuses on psychological phenomena as experienced and enacted by individual persons in their interaction with other persons and other parts of the environment, and in their development over time. Population-level research focuses on populations of individuals, frequencies of various psychological phenomena in a population, risk factors, and population-level effects of various psychological interventions. Mechanism-level research focuses on psychological functioning as explained in terms of neurophysiological mechanisms and information processes at a sub-personal level. It is argued that the failure to differentiate clearly between research questions at these three levels lead to questionable research practices. Most notably, a failure to differentiate clearly between the population level and the person level leads to problem-method mismatches in the form of researchers trying to answer questions about persons by research on populations. Also, because of a failure to differentiate between the person level and the mechanism level, explanations in terms of sub-personal mechanisms are too often seen as providing answers about what occurs at the person level, thereby failing to study persons as intentional agents in interaction with other persons and other parts of the environment. It is argued that a clear differentiation between three levels of psychological science – population, person, and mechanism – may contribute to an increased clarity in these matters and may thereby contribute to the development and maturation of psychological science.

## Introduction

There are many possible ways of dividing psychology into subdisciplines. The purpose of the present paper is to explore one specific meta-perspective on psychological science, seen as having three main branches: *person* psychology, *population* psychology, and *mechanism* psychology. The basic suggestion is that psychological science involves research at three different levels: (1) a person-level, (2) a population-level, and (3) a sub-personal mechanism level. The person-level is characterized by a focus on psychological phenomena as experienced and enacted by individual persons in their interaction with other persons and other parts of the environment, and as developing over time. The population level involves a focus on populations of individuals, the frequency of various psychological phenomena in a population, and population-level effects of various psychological interventions. At the sub-personal mechanism level the focus is on explaining psychological functioning in terms of neurophysiological mechanisms and information processes.

This suggestion is inspired by a division that is commonly made in genetics. The study of genetics (as described, for example, by Pierce [2020]), consists of three major subdisciplines: classical genetics (transmission genetics), population genetics, and molecular genetics. In classical genetics, which is also known as transmission genetics, the focus is on the *individual organism*, and how an individual inherits its genetic makeup and passes its genes to the next generation. In population genetics the focus is on the group of genes found in a *population*, that is, the genetic composition of populations of individuals, and how that composition changes geographically and with the passage of time. Finally, in molecular genetics the focus is on *molecular processes* within the individual, such as cellular processes of replication, transcription, and translation (by which genetic information is transferred from one molecule to another) and gene regulation (the processes that control the expression of genetic information). In other words, this implies a three-level model, where the three levels are represented by individuals (classical genetics), population of individuals (population genetics) and sub-individual processes (molecular genetics).

**Figure 1:**
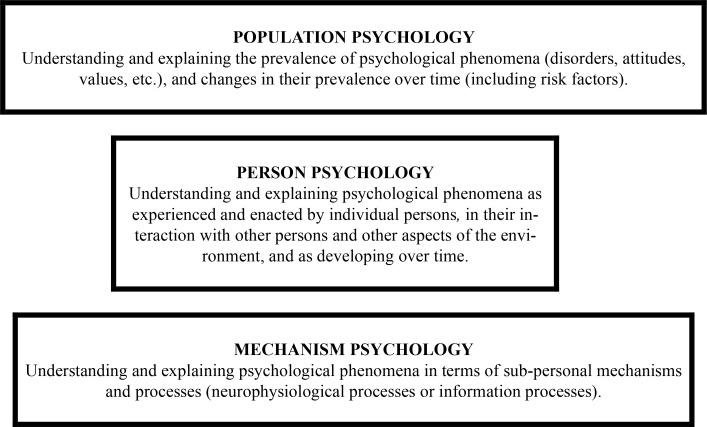
The three branches of psychological science as differentiated in terms of the type of understanding strived for.

The basic argument in the present paper is that a similar three-branch model of psychological science can produce increased clarity concerning research questions and research methods and how they fit together. Population-level and mechanism-level research represent opposite poles of psychological science – the former focusing on *populations of* individuals, and the latter on *mechanisms within* individuals. In between, there is the level of individual persons and their experiences and intentions, their interaction with other persons and other part of the environment, and as changing and developing over time. Research questions at these three different levels require *research methods that match the respective research questions*. A failure to differentiate clearly between research questions that belong to these different levels lead to mismatches between research questions and methods, and to ill-founded research strategies. Figure 1 gives an overview of the different kinds of understanding that are strived for in these three different branches of psychological science.

Although the person-level might be expected to form the core of psychology, it has a rather marginal role in present-day psychological research. Several writers have asked why this is so and have argued for a more central role of the person in psychological research. For example, in 1971 Rae Carlson asked, “Where is the person in personality psychology?” And in 2004, Peter Molenaar published a manifesto for “bringing the person back into psychology”. Still to the present day, however, the person has a rather peripheral role in psychological science, which is dominated either by a focus on populations of individuals or on mechanisms and processes at a sub-personal level.

The present paper has two main sections. The first section focuses on the differentiation between population- and person-level research. This is done in terms of a brief review of three areas of research: (1) epidemiological research and survey research (which clearly belong to the population level); (2) correlational research (where mismatches are found between research questions formulated at the person-level and the use of research methods at the population-level); and (3) psychotherapy research (where population-level methods dominate heavily at the cost of the kind of person-level research that is required to understand how psycho-therapy works).

The second section focuses on the differentiation between person- and mechanism-level research. Focus is primarily on two subareas within mechanism-level research: neuro-science and information processing psychology. It is argued that, although research in these areas is essential to the understanding of human psychological functioning, findings from this research are insufficient to understand psychological processes at the person-level. The reason for this is that the latter requires a focus on persons considered as intentional agents in interaction with other persons and other parts of the environment.

## Differentiating the Population Level and the Person Level

The beginnings of empirical psychological science are generally dated to the last part of the 19th century, and the German researcher Wilhelm Wundt is commonly mentioned as one of the pioneers. At that time, however, the individual was still the primary focus of research. As described by Danziger (1990) in his study of the early history of psychological research, virtually all studies that were published in experimental psychological journals before World War I attributed the results to specific individual subjects, who were referred to by name, letter, or initials. This style of research, however, declined rapidly during the first part of the 20th century. Instead, an alternative style of research ascended, which focused on populations of individuals, rather than on individual persons. According to Danziger (1990), this new style of research had its roots in the development of social statistics on public health, crime, suicide, poverty, etc., and was closely linked to the use of questionnaires as tools for investigation.

Three different varieties of population-level research will be briefly discussed in the present section: (1) epidemiological and survey research, (2) correlational research; and (3) the use of randomized controlled trials (RCTs) in psycho-therapy research. It is argued that, whereas epidemiological research as well as survey clearly belong at the population-level, and seldom involve any mismatches between research questions and research methods, the situation is different with correlational research and psychotherapy research. The basic argument is that the two latter varieties of research involve major mismatches in the form of person-level questions that are addressed with population-level methods.

### Epidemiological Research and Other Survey Research

It is generally recognized that certain kinds of research questions demand a focus on populations. This is true of all research questions that have the form “How common/frequent/prevalent is…?” These kinds of questions may be asked, for example, about mental disorders, attitudes, beliefs, and values. One typical example is epidemiological research, which is the systematic study of the distribution (frequency, pattern) and associated factors (predictors, risk factors) of health-related states and events in specified populations. Typical research questions in this kind of research ask for *percentages of individuals* who have a certain condition at a certain time (point prevalence) or during a certain period (e.g., one-year prevalence) or during life (life-time prevalence), or who develop a certain condition during a particular time period (incidence). Other important research questions concern the identification of predictors and risk factors for the development of various disorders. The term “predictor” often means nothing more than a correlate, whereas the identification of risk factors requires the documentation of temporal precedence. That is, the elevation in the risk factor must be there before the change in the outcome variable, otherwise it is merely a correlate (e.g., Kraemer et al., 1997).

In rare instances it is possible to study the full target population. Almost always, however, the target population is too large, and a representative sample needs to be selected for study. The methodology then involves the use of various techniques (e.g., random sampling) to get access to samples that are representative of the population of interest. The purpose is to draw general conclusions about populations of individuals from the results of studies with representative samples, and the various problems that can get in the way of this are generally referred to as threats to the external validity of the conclusions (e.g., Shadish et al., 2002).

Population-level research is also relevant in many other research areas where the research question has the form “How common/frequent/prevalent is…?” One example is the research done with the World Values Survey (e.g., Inglehart et al., 2008). This research has studied the prevalence and change over time of (1) traditional values versus secular-rational values and (2) survival values versus self-expression values in different parts of the world.

It is difficult to conclude otherwise than that population-level research is directly relevant when one is searching for answers to questions about how *frequent* or prevalent something is, or when one is searching for predictors and risk factors. It is difficult to imagine that this kind of research could possibly be replaced by some other research approach. In other words, here we have a clear example of a *match* between research question and method. There are, however, other forms of population-level research that can be questioned in this regard. One of these is psychological research with correlational designs, and another is the use of randomized controlled trials (RCTs) in psychotherapy research. Here various problems and complications have been pointed out, and several kinds of criticism have been raised.

### Correlational Psychological Research

In epidemiological and other survey research the primary focus is on questions about prevalence (e.g., of mental disorders or attitudes) in populations of individuals. Although individuals are here characterized in terms of *variables* (e.g., dichotomous variables in the form of presence or absence of a certain disorder, or a certain attitude), the focus is still on populations of *individuals*. This is also the case in studies using cluster analysis or other person-oriented methods used to classify individuals in terms of *profiles* or *types –* although the individuals studied are characterized in terms of patterns of values on *variables*, the focus is still on subgroups *of individuals*.

In correlational psychological research, however, the focus changes from populations of individuals to relations between values on variables in the population (as measured, for example, by various correlational methods, multiple regression, or structural equational models). In short, the *relation between variables in populations of individuals* here replaces the *populations of individuals* as the focus of research. In research with longitudinal design this is motivated by a shift from research questions about *prevalence* to research questions about the *causal processes* involved in individual development. But a basic question here is to what extent this methodology really matches the research questions.

As argued by Richters et al. (2021), longitudinal studies which use this kind of methodology often start by formulating their research questions in terms of individual persons and their characteristics, and then subtly change focus onto data at the aggregate level, before they finally return to discuss the findings again at the level of individuals. Richters (2021) examined a selection of 39 longitudinal research studies from eleven leading psychology journals (selected because they were published in the first 2019 issue of these journals) and found a typical pattern in these studies. He describes how all of these papers (1) start by formulating research questions in terms of the *psychological characteristics of individual persons*, and then (2) “abruptly and without comment” (p. 371) shift focus to *statistical properties of data at an aggregate level*, to (3) finally in the Discussion section, “seamlessly and without explanation” (p. 371) shift the unit of analysis back again from the aggregate level to the individual level.

What, then, is the problem with shifting focus in this way, from formulating the research question in terms of individual persons to using a variable-oriented analysis at an aggregate level to answer the research question? The basic problem, according to Richters (2021), is that there is an irreconcilable *mismatch* between what he refers to as the *psychological homogeneity assumption* of this research paradigm^1^ and the *psychologically heterogeneous* character of the phenomena under investigation, which makes the methodology “intrinsically incapable of advancing theoretical knowledge about the causes of psychological and behavioral phenomena” (p. 366).

#### The problematic assumption of psychological homogeneity

As an illustration Richters (2021) takes a bivariate correlation of .34 between marital discord and antisocial behavior.^2^ There are at least seven different possible explanations of such a correlation. For example, marital discord may contribute to the development of antisocial behaviour, either (1) directly or (2) indirectly (i.e., via changes in some other variable). Or antisocial behaviour may contribute to marital discord, either (3) directly or (4) indirectly (i.e., via changes in some other variable). Or both processes may be at work at the same time, forming a reciprocal causal relationship between the two variables, either (5) directly or (6) indirectly (i.e., via changes in some other variable). Or (7) the correlation may be spurious in the sense that marital discord and antisocial behavior are causally unrelated, being an artifact of the influence of some third variable on each. A basic problem with research within the psychological homogeneity paradigm is that it starts from the assumption that *one of these explanations is the correct one, and that the task is find out which*. More specifically, the assumption is “that these 7 possibilities are also mutually exclusive conditions. That is, they are genuinely competing for explanations for the .34 correlation in the sense that only one can be correct” (Richters, 2021, p. 385).

This is the psychological *homogeneity* assumption at work. It implies that one of the theoretically possible causal structures applies to the entire population in question (i.e., equally to all individuals). According to Richters (2021), however, it is much more likely that what applies here is psychological *heterogeneity*, in the sense that the causal relation between marital discord and antisocial behaviour may look *quite different in different cases* and may be entirely absent in some cases. He describes several possible scenarios of how antisocial behavior may be related in various ways with marital discord in different social environments and contexts, including the possibility of a reverse association in some cases (i.e., where antisocial behavior serves to reduce marital discord).

In other words, it is highly likely that there are several different kinds of causal associations and noncausal associations between marital discord and antisocial behaviour *which apply to different subsets of individuals*. In this perspective the *r*=.34 correlation is “a statistical artifact, a theoretically uninterpretable residue of multiple, qualitatively different causal structures” (Richters, 2021, p. 386). At best this correlation might indicate a “causal influence of marital discord on antisocial behavior that holds for only a subset of sample members” (p. 387), although “artifacts stemming from unrecognized heterogeneity can just as easily give rise to an illusory .34 correlation in the absence of a causal influence of marital discord on antisocial behavior for even a single sample member” (p. 387).^3^

These kinds of phenomena are not new and have been recognized under various terms such as the ecological fallacy (Robinson, 1950), Simpson’s paradox (Simpson, 1951), and non-ergodicity (Molenaar, 2004). The ecological fallacy is the interpretation error that occurs when inferences from groups are inappropriately generalized to individuals, whereas Simpson’s paradox refers to the situation when trends in subgroups differ from (or are even inverse to) the aggregate trend in the whole group. Non-ergodicity implies a lack of generalizability from group statistical estimates to individual statistical estimates, and that “only under very strict conditions – which are hardly obtained in real psychological processes – can a generalization be made from a structure of interindividual variation to the analogous structure of intraindividual variation” (Molenaar, 2004, p. 201).

In the case of bivariate correlations, such as the *r*=.34 correlation between marital discord and antisocial behaviour that is discussed by Richters (2021), the simplest way of approaching these questions is to inspect the scatter plot, which provides individual-level information about the correlation. Some rank correlations^4^ also include information on how many individuals in a sample behave in the way the sample’s inter-individual correlation suggests. To establish what Fisher et al. (2018) refer to as *group-to-individual generalizability*, however, we need to collect intraindividual data over time and compare the *intra*individual variation in such data with the *inter*individual variation in data from cross-sectional measurements of individuals at one time point. As Fisher et al. (2018) points out, such generalizability has not yet been systematically examined. This poses

a threat to human subjects research, because we do not know the full scope of the problems and are not adequately studying it… Hitherto, the highest-impact publications in medical and social sciences have been largely based on data aggregated across large samples, with best-practice guidelines almost exclusively based on statistical inferences from group designs. The worst-case scenario—a global, uniform absence of group-to-individual generalizability due to nonergodicity in the social and medical sciences—would undermine the validity of our scientific canon in these domains. However, even moderate incongruities between group and individual estimates could result in imprecise or potentially invalid conclusions. We argue that this possibility should be formally tested, wherever possible, to be ruled out. (Fisher et al., 2018, p. E6106)

Moeller (2021) similarly speaks of the risk for a new credibility crisis comparable to the replicability crisis, “because we have only started to understand how many of the conclusions that we tend to draw based on between-person methods are based on a misunderstanding of what these methods can tell us and what they cannot” (p. 53).

What, then, do the limited data available say about group-to-individual generalizability? Fisher et al. (2018) utilized data from six different samples to explore this question. Their conclusion was that this kind of generalizability may be worryingly imprecise. The variance was found to be up to four times larger in individuals than in groups, and the average intraindividual correlations differed clearly from the corresponding interindividual correlations. To take one of the more striking examples: whereas only positive *inter*individual correlations were found between fear and avoidance, the corresponding *intra*individual correlations were much more varied, with one subgroup showing no correlation and a small portion of individuals even exhibiting a negative correlation – that is, they appeared to be more likely to approach a situation when experiencing fear.

There is empirical evidence of non-ergodicity also in other areas. Molenaar and Campbell (2009), for example, factor-analyzed intensive longitudinal data from single individuals on a questionnaire measuring the Big Five personality factors and found that the number of personality factors varied, with different individuals exhibiting two, three, or four factors. Moreover, the intraindividual models did not differ only in the number of factors but also in how the factors related to the items, as expressed in the patterns of factor loadings. They concluded that “the nominal interindividual (Big Five) structure cannot be generalized to the level of variation within each subject” (p. 115).

Ergodicity has also been tested in the field of cognitive abilities. To test if the well-established hierarchical model of intelligence with its *g* factor could be identified also on the level of the individual, Schmiedek et al. (2020) analyzed the structure of cognitive abilities in 101 young adults performing nine cognitive tasks on 100 occasions distributed over six months. Their results showed that the structures of individuals’ cognitive abilities showed a lot of variation and deviated greatly from the between-person structure. Although working memory contributed the largest share of common variance to both between- and within-person structures, the *g* factor was much less prominent within than between persons. They concluded that to reveal the development and organization of human intelligence, individuals need to be studied over time.

#### Causality at the population level and causality at the person level

As shown by the preceding examples, it is difficult to generalize from data at the group-level to the individual. How then should we view correlational findings at the population level? Some critics have questioned that this kind of research even belongs to psychology. Lamiell (1998), for example, argued that the establishment of general principles of personality does not need data on individual differences in aggregates of individuals but data about basic processes of individual development (i.e., data within individuals over time). He went even further to suggest that study of individual differences in aggregates of individuals “is much less a *psychology* than a *demography* exploiting a psychological vocabulary” (p. 34). Here it may be argued that Lamiell partly misses the mark. An alternative view is that it is quite meaningful to speak of psychological knowledge *both* at the population level and at the level of the individual.

It may, for example, be quite meaningful to analyze both between-person and within-person correlations even when they go in different directions. This is illustrated by Hamaker’s (2012) example of the correlation between typing speed and errors among typists. Here it seems quite reasonable to find a *negative* correlation at the population level in combination with a *positive* correlation at the person level. At the population level, we have individuals with varying levels of typing expertise, where those with higher expertise are likely *both* to type faster than those with low expertise *and* to make fewer errors – thereby producing a *negative between-person correlation* between typing speed and errors. At the person level, however, it is to be expected that faster typing will be associated with more errors, thereby producing a *positive within-person correlation*. One reason for this is that in this case the *causal structure* differs between the population level and the person level: at the population level typing expertise is a causal variable, whereas it is not at the person level (the degree of expertise being constant within each person, at least in the short run). This shows that, at least in some cases, correlations at both levels are equally meaningful and informative, even when they go in opposite directions.

Another classic example of discrepancies between correlations at the population level and the person level (although the correlations do not go in opposite directions in this case), as described by Asendorpf (2015), is

the correlation between angriness and happiness (population level) versus the correlation between being angry and being happy (person level). In diary studies where participants report the intensity of emotions in particular situations (see already Epstein, 1983), angriness (the average report of being angry across all situations) and happiness (the average report of being happy across all situations) correlate only slightly negatively because of interindividual differences in the overall tendency to report intense emotions (some “unemotional” participants report both low angriness and low happiness, others report both high angriness and high happiness). In contrast, being angry and being happy correlate strongly negatively across situations within persons because situations where one experiences mixed angry-happy emotions are rare. (Asendorpf, 2015, p. 49)

Again, both correlational findings are equally “real”, and the discrepancy between them can be explained by different causal structures being operative at the person level versus the population level. At the population level degree of emotionality (or affect intensity as a trait) enters as a causal variable that differs between individuals, whereas at the person level this can be regarded as constant, at least in the short run.

Similarly, questions about causal relationships can be pursued meaningfully both at the population level and at the person level, because these are *different kinds of questions*. Consider for example the case of non-suicidal self-injury (NSSI; e.g., Nock, 2009). At the population level, the research question might be “Why has NSSI increased among adolescents during the last decades?” This asks for a causal explanation of changes in the prevalence of NSSI, which may involve social and cultural factors. At the person level, in contrast, the research question is about the causal factors in an individual’s life that make them engage in NSSI. In this example we are dealing with causal processes over different lengths of time: The factors that cause changes in the prevalence of NSSI operate over a span of years and decades, whereas the factors that cause the individual to engage in NSSI probably operate at a span of days and hours.

There are indications that not only NSSI but also eating disorders and other aspects of psychological distress have increased among young people during the last decades. Studies at the population level may lead to important knowledge about the nature of such changes and the causal factors involved. Even if this kind of research should not be of much help to understand the causal factors involved at the level of the individual, it still represents an important form of psychological knowledge.

This is not to say that all correlational studies at the population level are meaningful. What is to be radically questioned is *the use of research designs from the population-level to answer questions about individual functioning and individual change*. Used for the purpose of answering research questions that belong to the population level, however, they are clearly motivated.

### RCTs in psychotherapy research

Psychotherapy research is a huge field with many different research questions and research methods. Much of present-day psychotherapy research, however, takes place at the population level. This is the case with so-called randomized controlled trials (RCTs) which are generally seen as the golden standard when it comes to establish evidence for psychological treatment methods. Here a central research question is about the causal efficacy of treatments and basically takes the form “how to treat a given disorder in the most efficient way?” This means that the kind of validity that is strived for is different from that in epidemiological research: here the most important form of validity is not *external* validity (i.e., drawing valid conclusions about the population of interest) but *internal* validity (i.e., drawing valid conclusions about the causal effects of specific treatments on individuals with various kinds of conditions; Shadish et al., 2002). Methodologically this means that RCTs do not require *random sampling* (which would anyway be difficult to implement in treatment studies, where participants must choose themselves if they want to be part of a study) but the *randomization* of participants to the treatments that are to be compared, so that other possible causal factors can be controlled as far as possible.

RCTs have shown to be of immense importance in medicine, where they have made it possible to evaluate the relative efficiency of various treatment methods and thereby help to improve medical practice. This methodology has also made it possible to identify ineffective and harmful treatments in various areas, even where the previous use of correlational designs had produced promising but illusory findings. To take one example, Bjelakovic et al. (2007) carried out a systematic review of RCTs that had compared high doses of antioxidant supplements such as beta-carotene with placebo and found no evidence of positive effects on health; in fact, the results even indicated negative effects in the form of an increased mortality. This was of quite surprising as observational studies with *correlational* designs had previously suggested that the use of antioxidant supplements was positively associated with healthy outcomes. Correlational findings of this kind, however, are always subject to alternative explanations. A positive association of this kind may, for example, occur if people who are healthy and more concerned about their health tend to consume more antioxidants than people who are less healthy from the start. RCTs do not suffer from these threats to the internal validity of the conclusions. RCTs have also for good reasons been suggested to be valuable for the detection of harmful psychological treatments (e.g., Lilienfeld, 2007).

The role of RCTs for the understanding of what makes psychotherapy work, however, is much more in doubt. Al-though a general finding from RCTs is that psychotherapy does have positive effects *on the population level* (i.e., significantly more patients benefit from treatment than from be-ing in the control group), this kind of research has not provided much knowledge about *what* works in psychotherapy. As was summarized by Kazdin (2007): “after decades of psychotherapy research we cannot provide an evidence-based explanation for how or why even our most well-studied interventions produce change” (p. 1). This conclusion still seems to hold.

#### RCTs in relation to the research question “What makes therapy work?”

It has been exceedingly difficult to find significant differences in effect between different forms of psychotherapy in RCTs. For example, although cognitive-behaviour therapy (CBT) is the by far most examined form of psychological treatment for depression and is recommended in most treatment guidelines, it has been difficult to show that it produces better effects than other psychological treatments. In the largest meta-analysis ever carried out of a specific type of psychotherapy for a mental disorder, Cuijpers et al. (2023) studied the effects of CBT for depression by including 409 RCTs (518 comparisons) with 52,702 patients. CBT was found to be more effective than control conditions such as usual clinical care and waitlist conditions. In terms of percentages, 42% of the patients in CBT responded to treatment, while the response rate was only 19% in the control groups. In the short term, the effects of CBT were comparable to those of pharmacotherapies, but at 6-12 months follow-up CBT was significantly more effective. The comparison to other psychotherapies, however, showed a very small differential effect which was not sufficiently robust to remain significant in sensitivity analyses.

These kinds of conclusions are representative of the field of psychotherapy research, and they are compatible with a wide variety of possible explanations. Among the possible explanations are, for example, (1) that the effects are due to factors that are *common* to different forms of therapy and that are present to an *equal* degree in the different treatments; (2) that the effects are due to *different* treatment components in different forms of therapy, although these different components are *equally effective* at an average for the patients in the different treatments; (3) that the effects are due to *different* treatment components for different patients even within each treatment condition, but that these different components are *equally effective* at an average (e.g., Lundh, 2014).

One main problem that makes it much more difficult to interpret the results of RCTs in psychotherapy research than in medical research is that psychological treatments differ in many ways from medical treatments. RCT designs are quite suitable when the treatment in question *involves one specific intervention*, and this is more commonly the case in medicine than in psychotherapy. In general, the treatments studied in psychotherapy research represent large *treatment packages* involving many different interventions and interactions over a considerable time period. This is quite different from testing the efficacy of a tricyclic antidepressant versus placebo in patients with depression; here the experimental condition contains one specific component, which makes it easy to know where to attribute the effects.

An important problem with RCT designs in psycho- therapy research is that they suffer from a *low degree of control* over the experimental manipulation – that is, what takes place in the treatment. The therapies that are tested in RCTs are not described in terms of observable treatments (as is common in other areas of experimental psychology) but in terms of certain *constructs* that are used to label *entire treatment packages* (e.g., “cognitive behavior therapy”, “short-term psychodynamic therapy”, “interpersonal therapy”, etc.), the principles and procedures of which are outlined in *manuals*. The extent to which a treatment package is implemented as intended is called *treatment integrity* (Perepletchikova et al., 2007) and is assessed in terms of the therapist’s adherence to the manual plus therapist competence. These assessments are usually done retrospectively by trained observers from video recordings of therapy sessions. This, however, is not sufficient to solve the problem of low construct validity (e.g., Lundh et al., 2016), and meta- analyses indicate no evidence that adherence to the manual is significantly associated with treatment outcomes (Power et al., 2022; Webb et al., 2010).

What is needed for the further progress in our understanding of what makes psychotherapy work is clearly something else than RCTs. A possible lead is seen in the findings by Power et al. (2022) that, although treatment outcome showed no association with the degree of adherence to the manual, it did show a weak association with therapist *competence* as assessed by independent observers. Furthermore, as pointed out by Power et al. (2022), the magnitude of the latter effects was similar to the effect size of factors such as therapeutic alliance, goal consensus, and empathy. This suggests that what is at stake here are various forms of therapeutic *skills*, of both a relational and methodological nature. The effects of such skills are difficult to study by means of experimental designs (as this would require the randomization of patients to skilled versus non-skilled therapists, which would raise practical as well as ethical problems). Apart from the need for another kind of research design, with a focus on therapist skills “in action” (which suggests research at the person-level) this kind of research would also require the development of “a reliable taxonomy of therapist skills, and procedures for operationalizing and measuring these” (Lundh, 2017, p. 76).

#### RCTs in relation to the question “How to treat this specific patient?”

From the clinician’s perspective, a basic limitation of RCTs in psychotherapy research is their inability to provide guidance for the practicing clinician who needs knowledge at the person-level. The practicing psychotherapist has to deal with individual patients, and the primary question then is what kind of treatment each patient is likely to benefit from. Even if RCTs show that several different forms of psychotherapy produce equivalent effects *at the population level*, this does not say anything about which patient is likely to benefit from which treatment. One suggested solution is attempting to identify patient variables that are predictive of response to treatment, and that can therefore be translated into real-world treatment recommendations (e.g., Cohen & DeRubeis, 2018). The Personalized Advantage Index (PAI), for example, aims to combine research data into multi- variable prediction models that can generate individualized predictions to help clinicians and patients select the right treatment. These kinds of approaches, which represent an attempt to apply findings from population-level research to person-level clinical practice, however, have not been very successful (Cohen & DeRubeis, 2018).

To summarize this part of the discussion: Population-level research on psychological treatments may answer broad questions about whether a treatment has positive or negative or null effects at a group level. It may provide information about average effects and about how many patients are likely to respond to treatment, such as for example that around 42% of the patients respond to the treatment of depression. This, however, does not answer the main question of interest for the clinician: How should I best treat *this* patient?^5^ Neither does it contribute to answering basic theoretical questions about what works in psychotherapy. To the extent that RCTs are used for addressing such questions we have examples of a *mismatch between research problem and method*. Such questions belong to the person-level and cannot be answered by research at the population level; what is needed to answer questions such as these is a person-oriented approach to psychotherapy research (for more specific arguments along this line, see Lundh & Falkenström, 2019).

### The analysis of individual change and development

To summarize, it is undeniable that research at the population level has an important role to play in many areas of psychological science, such as psychopathology, psychotherapy research, social psychology, and cultural psychology. For example, research at this level can give us knowledge about how large a percentage of patient are likely to respond to a certain kind of psychological treatment, although it is not likely to provide information about *who* is likely to benefit from a certain treatment, or *what* makes a certain treatment work. Research at the population level is also likely to increase our knowledge about large-scale psychological changes that occur over time in a certain society or culture, as for example changes in values and attitudes, and changes in the prevalence of various kinds of psychological disorders. Problems arise, however, when research methods belonging to the population level are used to answer research questions at the person level.

Methods for analyzing development and change at the person level are required both in clinical psychology, where there is a need to understand the nature of change during various forms of psychological treatments, and in developmental psychology. As emphasized by Magnusson (2001), an individual’s developmental processes are characterized by continuous change and adaptation in a highly idiosyncratic way, which means that they “must, in the final analysis, be analyzed at the level of the individual” (p. 160). This requires methods for the study of *within-person change*. Van der Gaag (2023) similarly speaks of

a mismatch between our typical research methods – group-level analyses – and a core aim of developmental science: understanding the development of individuals. The implications are profound. Without insights into within-individual processes, our understanding of development remains incomplete and perhaps even incorrect, which could hinder the design of effective interventions. (van der Gaag, 2023, p. 1)

One approach to the study of individual development is the use of intensive longitudinal data, where idiographic data are collected by means of experiential sampling methods (ESM) or other forms of diary methods or ecological momentary assessment (EMA) (Csikszentmihalyi et al., 1977, 1987). This makes it possible to study intra-individual variation and change based on many repeated measurements. To take an example, Boswell et al. (2014) carried out an idiographic analysis of change processes in a patient with depression and anxiety who underwent unified transdiagnostic CBT treatment. The results showed, among other things, that changes in mindfulness and cognitive reappraisal preceded changes in depression and anxiety, and that the changes in mindfulness and reappraisal were most strongly associated with the stages of the treatment where the corresponding skills were trained. The authors concluded that the functional relationships found in this case should be made subject to systematic replication to see whether these results generalize over multiple individuals.

The latter is important. To advocate methods for the analysis of individual change and development is not to stay at the level of the individual person without any ambitions of theoretical generalization. Even though the process starts with individual persons, the findings naturally raise questions about the extent to which similar patterns recur from one individual to another. Comparing patterns between different individuals may then serve to develop the theoretical understanding even of the individual case. As Hayes et al. (2019) put it:

In order to understand why and how changes happen in an individual, we need to study the processes of change at the level of the individual, and then to gather nomothetic summaries based on collections of such patterns. (Hayes et al., 43)

Charbonnet and Conzelmann (2023) similarly emphasize the importance of starting the analysis of development “from the bottom-up—from the particular to the general—by keeping idiographic focus as long as possible” (p. 64). Here they differentiate three levels: (1) the individual person; (2) categories/subgroups (types) of persons with similar patterns; and (3) people in general (i.e., the population).

The “intermediate level” between the individual and the population at large is of special interest in the present context, as it can be approached *both* from a population perspective *and* from a person perspective. At the *population*-level of research, it is of clear interest to study how common various patterns are, and how large the various subgroups are. At the *person*-level, however, other more theoretical questions come into focus (e.g., about the causes and effects of these different patterns in different individuals); at this level the relative size of the subgroups with different patterns is of less interest. In fact, patterns that are relatively rare (perhaps shared by only a few individuals) may be of equal interest (and sometimes even larger interest) than patterns that are quite common (even if they are shared by a majority of individuals).

## Differentiating the Person Level and the Mechanism Level

The other large branch of psychological research besides population-level research is mechanism-level research. Here the basic research questions have the form “How to explain psychological functioning (e.g., perception, memory, learning, emotion, psychological development) in terms of underlying (neurophysiological or information processing) mechanisms?” This search for mechanisms is pursued in at least two large fields: neuroscience and information processing research. The former focuses on the brain and searches for neural mechanisms underlying psychological functioning, whereas the latter focuses on internal processes and mechanisms conceptualized in terms of information processing. Common to both approaches are their focus on sub-personal processes and mechanisms.

The purpose of this section is to discuss the difference between person- and mechanism-level of research, and to discuss some examples of research where this differentiation is not clearly made. This part of the paper is divided into four sections. In the first section, some examples are discussed of how researchers sometimes recognize that population-level research is insufficient but then search for a remedy in mechanism-level research, *without considering processes at the person level*. In the second section, the analysis of individual change processes at the mechanism level is contrasted with an analysis of individual change processes at the person level; whereas the former searches for mechanisms of change at a *sub-personal* level, the latter searches for *person-environment interactions* that are likely to lead to change. In the third section, it is argued that person-level and mechanism-level research are not competing but *complementary* strands of research. Finally, in the fourth section, the mereological fallacy is discussed as a misguided attempt to *model internal mechanisms after persons*, thereby attributing properties of persons to internal mechanisms.

### Mediators and Mechanisms – From Population-Level to Mechanism-Level Research

Sometimes when researchers want to include the individual in the equation, they move from the population level directly to the mechanism level, without paying much attention to the person level. To use psychotherapy research as an illustration, this is seen, for example, in Kazdin’s (2007) discussion of what is missing from population-level psychotherapy research (as exemplified by randomized controlled trials):

There has been enormous progress in psychotherapy research. This has culminated in recognition of several treatments that have strong evidence in their behalf. Even so, after decades of psychotherapy research, we cannot provide an evidence-based explanation for how or why even our most well studied interventions produce change, that is, the mechanism(s) through which treatments operate. (Kazdin, 2007, p. 1)

Kazdin’s (2007) attempted remedy is to focus on the identification of *mediators* and *mechanisms*. A mediator is “a construct that shows important statistical relations between an intervention and outcome, but may not explain the precise process through which change comes about” (p. 3). According to Kazdin’s reasoning, to explain the process responsible for change we need knowledge about the mechanisms at work. A mechanism is defined as “the basis for the effect, i.e., the processes or events that are responsible for the change; the reasons why change occurred or how change came about” (Kazdin, 2007, p. 2). And to provide evidence that a certain mechanism is responsible for therapeutic change, he argues that we need to establish the timeline which shows that *the mechanism changes before the symptoms.*

One of the examples of a hypothetical mechanism that is discussed by Kazdin (2007) is cognitive change in the cognitive treatment of depression. Based on evidence indicating that symptom change can occur before cognitive change, he argues against cognitive change as a likely mechanism. The validity of this conclusion is not what is at stake here. The main point is that the mechanism discussed refers to internal cognitive processes in the patient (i.e., *changes in information processing*). This is typical of research which stays at the mechanism level, without any attention to processes in the interaction between therapist and patient that would include the person-level in the picture.

Another example of a possible mechanism discussed by Kazdin is of a neurophysiological kind: the activation of critical receptors in the amygdala in connection with the exposure treatment of anxiety. He refers to evidence in support of this mechanism from experimental studies that show im-proved treatment effects when the critical receptor is activated by the oral administration of D-cycloserine 2-4 hours before each session (Kazdin, 2007, p. 14). Again, the main point for the present discussion is not the validity of this specific conclusion but the *absence* of any reasoning about what transpires in the *interaction* between therapist and patient during treatment. Again, this is research at the mechanism level which fails to include processes at the person level.

Interestingly, this failure to attend to the person-level does *not* mean that Kazdin (2007) ignores the value of within-individual data. He advocates a research design that includes the “assessment of mechanisms and outcomes all or most treatment sessions” (p. 18). The purpose is to provide a fine-grained analysis of *when* change takes place in both mechanisms and symptoms and how this differs from one individ-ual to another. As he takes care to point out, “patients in the same treatment conceivably could respond for different reasons” (p. 23), while at the same time repeating that “[u]nder-standing mechanisms of treatment is the path toward improved treatment” (p. 23).

In other words, the individual is clearly part of the picture here, but *not as a person in interaction with a therapist*, but rather as a “depository” of diverse mechanisms. Treatment is pictured as the delivery of interventions to a patient who responds in accordance with certain mechanisms. Although these mechanisms may differ from one individual to another, the person as an active purposeful agent in interaction with a therapist is no part of the picture.

A real move to the person-level requires a re-focusing to another set of research questions. These questions are about (1) *the therapist as a person* (e.g., with their professional and relational skills as enacted in the treatment), (2) *the patient as a person* (e.g., with some degree of motivation to engage in various parts of the treatment, such as homework tasks and a willingness to approach anxiety-evoking situations during exposure exercises), and (3) the *interpersonal inter-action* that takes place between therapist and patient (including their “person chemistry”). The main point is that *research at the person level requires more than merely a turn to intensive longitudinal data to investigate changes in individual patients.* A refocus of attention to the person-level requires a study of the interaction between two individuals in interaction, where each individual is seen as “an intentional, active agent in the interaction process” (Magnusson, 1988, p. 24). That is, therapist and patient are to be seen as intentional agents in interaction.

Although the person in this sense is missing from much psychotherapy research, some psychotherapy researchers have strongly emphasized the importance of studying patient and therapist as persons who operate on the basis of intentions and experiences. With regard to the patient, for exam-ple, Bohart (2000) has argued that “clients are active agents who operate on therapist input and modify it and use it to achieve their own ends” (p. 133), and that “clients are capable of using many different therapy approaches to resolve their problems…. However, they need some assistance: They have come to therapy because they have not solved problems with the resources available in their life spaces” (p. 133-134). This is also consistent with Seligman’s (1995) finding that clients who report being actively involved benefit most from therapy. The important point here is not whether Bohart and Seligman are right or not, but that his reasoning gives an illustration of hypothetical processes *at the person-level*.

Another illustration of person-level processes is given by Stiles’ (2013) concept of *responsiveness* as characterizing all kinds of human interaction. Examples at the most basic level are that people normally answer each other’s questions, stay on related topics, and take turns when they are speaking. In psychotherapy this means that the therapist does not just “deliver an intervention” but responds to the client’s behavior on a wide range of time scales. This also means that the technical procedures in a treatment package as described in manuals are typically carried out *in many different ways*, not only depending on the therapist’s personality and professional skills, but also as an adjustment to the patient’s personality and behavior. Further, what is of interest in psychotherapy is not any kind of responsiveness but *appropriate* responsiveness, aimed to achieve optimal benefit for the client. Again, this gives an illustration of processes at the person-level of analysis. Together all these examples illustrate the importance of differentiating between change processes at the mechanism-level and change processes at the person-level.

### Processes of Change at the Mechanism-Level and at the Person-Level

Hayes et al. (2019) have criticized the search for explanations of what occurs in psychotherapy in terms of mediators and mechanisms, as this implies “a simplistic, unidirectional input-output model” (p.43) which ignores feedback loops and bidirectional relationships, and is furthermore based on the use of statistical analyses (e.g., analyses of variance, regression models, and structural equation models) which assume generalizability from group data to the individual. Instead, Hayes et al.’s (2019) approach is to focus on *processes of change* at the level of the individual. More specifically, they advocate a *process-based* therapy approach based on the identification of change processes and the ambition to organize these into “a kind of multi-dimensional multi-level ‘functional periodic table’ for change processes” (p. 48).

In the present perspective, it is imperative to combine such an approach with a clear differentiation between change processes at the *person*-level and change processes at the *mechanism* level. Different research questions are involved at these two levels. Consider, for example, the exposure treatment of anxiety disorders (e.g., Abramowitz et al., 2011). At the person-level, it is essential that the patient actively engages in exercises that involve approaching some kind of anxiety-evoking situation (or anxiety-evoking thoughts, feelings or memories) that has previously been avoided, often in a graded procedure (starting with less anxiety-evoking situations and proceeding to more anxiety-evoking ones) as suggested by the therapist. The therapist contributes to the process by developing an analysis of the patient’s problem, providing the patient with a rationale for the treatment, and instructing and encouraging the patient to engage in the exposure exercises. A common instruction is to ap-proach what is habitually avoided, and to stay with the situation until the anxiety subsides. Various kind of relational and technical skills are relevant to make this into a successful process of change. An important research question at this level is how to arrange the therapist-patient interaction in the most efficient manner.

At the mechanism level, however, an entirely different set of questions enter the scene: questions about cognitive, emotional, and physiological processes of change that occurs at the sub-personal level. Many different theoretical concepts have been suggested by various researchers to capture the changes that occur at the mechanism level in successful exposure treatment. Some examples are “habituation”, “reciprocal inhibition”, “cognitive restructuring”, and “emotional processing” (e.g., Foa & Kozak, 1986). Importantly, the study of treatment outcome typically belongs to the person level, as long as it is based on measures of the patient’s experiences or overt behaviour. Research at the mechanism-level, however, has also shown evidence of changes after treatment. Studies using brain imaging, for example, show evidence of functional changes in the amygdala and anterior corticolimbic brain circuits after exposure treatment (e.g., Nechvatal & Lyons, 2013).

### Person-Level and Mechanism-Level Research – Not Competing but Complementary

Cognitive psychology is commonly defined as the scientific study of psychological processes such as perception, attention, memory, problem solving, reasoning, decision-making, etc. There are many varieties of cognitive psychology, of which some operate primarily at the person-level (e.g., Beck, 1976), whereas others operate at the mechanism-level. Among the latter is the information processing paradigm with its assumption that cognitive systems are essentially computational-representational systems (similar to computer software) that are processing input and generating output (behaviour) by information processing.

Critique of the information processing paradigm has come from many quarters, including proponents of ecological psychology (e.g., Gibson, 1966, 1979). Common to these criticisms is that this paradigm does not take sufficient account of the importance of *the interaction between the individual person and its environment*. By focusing only on processes that occur *within* the individual the information processing approach fails to give a fully relevant picture of psychological functioning. One of Gibson’s main contributions is the demonstration of the rich information that is available in the pattern of light which is reflected from each structured environment, and that converges in potential observation points in the surrounding medium (air or water) – a discipline referred to as ecological optics – and that may be sampled by living individuals who move around in that environment, provided that they have sufficiently sophisticated perceptual systems to pick up the available information.

Importantly, what Gibson points to here is the information that is available *in the environment* (“stimulus information”) as distinct from the neural or cognitive processing of such information. But he also questions the very need for cognitive processing of this information. What is required, he says, is rather a perceptual system that is “attuned” to this information and can “resonate” to it. As Gibson (1966, p 271) puts it, “a system ‘hunts’ until it achieves clarity”, a clarity that consists in a kind of “resonance” of the system to incoming information. Here, however, he tends to go too far in his criticism. What we have here are *theories at different levels*: Gibson’s ecological theory belongs to the person level, whereas the information processing paradigm belongs to the sub-personal level of mechanistic psychology. Ecological psychology may contribute to an understanding of how individuals are continuously connected with and interacting with their environment (which includes the pick-up of information from the environment), whereas information processing research can help us understand the processes that occur *within* individuals when they perceive, think, remember, etc.

One example of an information processing theory is Baddeley’s (2012) model of working memory, which contains four components: (1) a *phonological loop*, where we hold verbal-accoustic information in store temporarily by subvocal rehearsal; (2) a *visuospatial sketchpad*, where we keep visual and spatial information in store temporarily by means of visuospatial imagery; (3) an *episodic buffer*, a multidimensional interface assumed to be capable of binding information into episodes that are then available for conscious awareness; and (4) the *central executive*, which controls the allocation of attention between information in the different memory stores, and manipulates the information available. This model is designed to explain various parts of psychological functioning, such as our ability to rehearse information (e.g., telephone numbers, the route we have walked, whole episodes, etc.) to keep it in mind temporarily, where it can be elaborated for various purposes. Among other things research based on such models can help to explain limitations in our ability to attend to and remember things.

The theoretically most problematic aspect of Baddeley’s model is probably his concept of “the central executive”. As Baddeley (2012) himself notes, the central executive is assumed to be “capable of attentional focus, storage, and decision making, virtually a homunculus, a little man in the head” (p. 13-14). In other words, here we have a hypothesized internal *mechanism* which is assumed to capable of things that we ordinarily attribute to *persons*, such as choosing where to focus attention and making decisions. Baddely recognizes the critique that he has received for taking this approach, but responds in the following way:

I regard homunculi as potentially useful if used appropriately. It is important that they are not seen as providing an explanation, but rather as a marker of issues requiring explanation. Provided the various jobs performed by the homunculus are identified, they can be tackled one at a time, hopefully in due course allowing the homunculus to be pensioned off. (Baddeley, 2012, p. 14)

Logie (2016) argues that the time has come to “retire” the central executive. He points to the problematic assumption of an inner homunculus that is supposed to control the allocation of attention and other aspects of thinking, which leads “to concerns about what is controlling the executive, what is controlling the controller, and so on, with the risk of having an infinite hierarchy of executive controllers or homunculi” (Logie, 2016, p. 2094). Instead, he suggests an explanation in terms of self-organizing principles from multiple local interactions, where “the level of activity of different brain structures and networks shifts dynamically between them according to the demands of the task, not the result of a single, centralised control mechanism” (Logie, 2023, p. 2453).

The important thing about this reasoning, in the present context, is that this suggests a possible *mechanism*-*level explanation* of the *person-level experience* of agency in attending, rehearsing, imagining, deciding, reasoning, etc. That is, it makes it possible to differentiate clearly between the person- and the mechanism-level in a way that opens up for the search for explanatory models that do not mix up these two levels in the way that is done by the concept of a “central executive”. Paying attention, deciding about where to focus attention, rehearsing things, imagining things, thinking, and reasoning are things that *persons* do. The ability to do all these things requires an explanation in terms of the internal mechanisms at work, but *it is a mistake to model these internal mechanisms on the functioning of persons*. If we model internal mechanisms on intentional agents, we are likely to end up in an infinite regress of inner persons (homunculi) of the kind described by Logie (2016).

### The Mereological Fallacy

Explanations in terms of a homunculus represents a kind of reasoning that Bennet and Hacker (2003) have referred to as “the mereological fallacy”. Mereology is the logic of part/whole relations, and the mereological fallacy has been defined as the mistake of attributing properties that belong the whole to a part of the whole. As argued by Bennet and Hacker (2003), “psychological predicates which apply only to human beings or (other animals) as wholes cannot intelligibly be ascribed to their parts, such as the brain” (Bennett and Hacker, 2003, p. 29). The mereological principle means that psychological capacities (such as perceiving, thinking, deciding, choosing, feeling, etc.) apply to human beings as *wholes* and should not be ascribed to their *parts*, such as the brain, parts of the brain, or some hypothesized internal component of information processing. Although Bennet and Hacker (2003) see it as an essential task for neuroscience to explain how perceiving, thinking, deciding, choosing, feeling, etc., is done, this must be done without losing sight of the fact that it is the *individual person*, not its brain, who does all these things.

Other examples of the mereological fallacy are seen in theoretical approaches that attribute *representations* to various parts of the brain. Taking the visual system as an example, critics have talked of several different theoretical fallacies here. One example is what Gibson (1966) referred to as the *retinal image fallacy*. The retina is the inner light-sensitive layer of tissue of our eyes, and the optics of the eye create a focused two-dimensional image of the visual world on the retina. The retinal image fallacy arises when people assume that what we see is this retinal image, and that visual perception occurs inside the brain as the result of a processing of the retinal image. Gibson (1966) referred to this conception of visual perception as the “little man in the brain” theory, implying that there is a little man, a homunculus, seated in the brain who looks at this physiological image. The homunculus would then have to be equipped with an eye to see it with, and so we would have explained nothing by this theory. We are in fact worse off than before, since we are confronted with the paradox of an infinite series of homunculi, each within the other and each looking at the brain of the next bigger one. (Gibson, 1979, p. 60). Natural vision, according to Gibson, takes place when we explore the environment by walking around and by moving the head to gather information about things; “natural vision depends on the eyes in the head on a body supported by the ground, the brain being only the central organ of a complete visual system” (Gibson, 1979, p. 1).

A partly similar argument was set forth by Skinner (1969), also directed at the fallacy of attributing visual perception to some kind of representation, this time deeper inside the brain:

Suppose someone were to coat the occipital lobes of the brain with a special photographic emulsion which, when developed, yielded a reasonable copy of a current visual stimulus. In many quarters this would be regarded as a triumph in the physiology of vision. Yet nothing could be more disastrous, for we should have to start all over again and ask how the organism sees a picture in its occipital cortex, and we should now have much less of the brain available in which to seek an answer. (Skinner, 1969, p. 232)

Representing things is something that *persons* do, for example when they mentally imagine, verbally describe, or draw something, but it is not something that should be ascribed to brains, or parts of brains. “The ’representation’ is a weed in the neuroscientific garden, and the sooner it is uprooted the better”, as Bennet and Hacker (2003, p. 143) put it. This does not in any way mean to deny that activity in the brain may serve as *correlates* of objects that are perceived and may thereby “represent” these objects in the simple sense that changes in the brain activity are caused by those objects. In this limited sense, however, the concept of representation has no explanatory value of the kind that cognitive neuroscientists require of it.

To summarize, mechanism level research plays an important role in psychological science. Because it deals only with what occurs *within* the individual, however, it can in no way replace person-level research. Neither should internal mechanisms be modelled on persons. To understand the interaction between the individual and the environment, and between individuals, we must turn to research at the level of the person. This, however, is not to deny the value of mechanistic psychology. *Person psychology and mechanism psychology operate at different but complementary levels of psychological research.*

## Discussion

The main thesis argued for in the present paper is that psychological science can be divided into three main branches: *person* psychology, *population* psychology, and *mechanism* psychology, corresponding to three levels of research. All three branches are important, but the person level is too often overlooked or ignored. It is also too often erroneously assumed that population- or mechanism-oriented research can substitute for person-oriented research. It is of paramount importance to differentiate the person-branch of psychological science from the two other branches, without denigrating any of these. The purpose of this concluding discussion section is to discuss some research questions at the person-level of research and how to approach these.

The kind of psychological theory that is required at the person-level is about individual persons as *wholes* (which requires a holistic person-oriented perspective), their *interaction* with the environment (which requires an interactional perspective), their *experiences* (which requires a phenomenological perspective), and their *interpersonal relations* (which requires a relational perspective). Similar conclusions have been expressed by many different writers, but the first to explicitly advocate a holistic-interactionistic paradigm were probably Bergman and Magnusson (1997; see also Bergman, 1998, 2001; Bergman & Andersson, 2010; Magnusson, 1999, 2001). Recent attempts to contribute to the development of a general theory of persons have been presented, among others, by Peter Ossorio (2006) and Christian Smith (2010).

The need for a general theory of persons is probably most acute within clinical psychology, where the purpose is to help people who suffer from various problems, which require an understanding of individual persons. Because all individuals with the same kind of diagnosis do not respond in the same way to the same kind of treatment, there is a need for a more detailed analysis of the individual case to decide about the optimal treatment. In psychotherapy (or at least in some varieties of psychotherapy) the treatment plan is based on a personalized analysis of the patient’s presenting problems. This is most explicitly seen in cognitive-behaviour therapy (CBT) which relies on various models for *case conceptualization* as the basis for developing a personalized treatment plan. An early variety of this is the functional analysis of behaviour, with its roots in Skinner’s (1969) theory of how an individual’s behaviour is formed and maintained by their interaction with environmental “contingencies of reinforcement”. Another variety is the cognitive conceptualization of the patient’s problem (e.g., Kuyken et al., 2009) in terms of how cognition, emotion, and behaviour interact in the individual case. More integrative forms of conceptualization, which integrate psychodynamic concepts, have been proposed by Eriksson (2014).

Importantly, these models of analysis belong primarily to the *person* level and not the mechanism level. One problem, however, is that there is little empirical evidence for the usefulness of these kinds of case conceptualization. What is required here are reliable methods for case conceptualization, based on well-founded theories about psychological change and development, persons-environment interaction, and interpersonal relations – and an empirical documentation that these kinds of case conceptualization matter, i.e., that they are associated with an improved treatment outcome.

A relatively new kind of case conceptualization is socalled network analysis. In this approach, psychological problems (e.g., a depression) are seen as elicited by some life event but then maintained by a network of symptoms that have effects on each other in a way that stabilizes the problem (Borsboom, 2017; Cramer et al., 2016). These networks are seen as highly idiosyncratic in a way that differs even between individuals with the same psychiatric diagnosis. The relations between the symptoms are graphically represented in networks where symptoms are shown as “nodes” and causal inter-symptom relations are shown as “edges”. One important aspect of such an associative network of symptoms, which may possibly explain why some individuals are more vulnerable than others, is the strength of its *connectivity*:

A weakly connected network will, under low external stress levels, occupy a stable state of mental health... The network is resilient because – even if it may feature symptomatology if put under stress… it will return to its stable state when that stress level diminishes. In contrast, a highly connected network may be asymptomatic… but is vulnerable because – as soon as a stressor arises in the external field – it can transition to an alternative stable state of mental disorder. (Borsboom, 2017, p. 10)

In this model the mental disorder is seen as an emergent state in a complex dynamic system. This exemplifies a line of thinking about individuals in terms of dynamic systems, seen as systems of self-organizing processes (Ashby, 1962). It remains to see if this kind of analysis can contribute to the improvement of psychological treatment. Still, even if this will be the case, this kind of network model is not about *persons* with psychopathology, but about psychopathology as associative *networks of symptoms*. That is, it belongs to the mechanism level rather than the person level. The person as an intentional agent in interaction with the environment is no part of the picture. The environment enters the scene only as events that cause an activation of the network; or as Borsboom puts it, “trigger events in the external field (e.g., adverse life events) produce network activation” (p. 8).

Among the methodological tools from the natural sciences that Magnusson (2001) thought to be useful for person-oriented research are nonlinear dynamic models. But he also emphasized that, if these models are to be useful, they must be adapted to the nature of the psychological processes that characterizes individual persons:

At that level, a fundamental characteristic and guiding element in an individual’s functional interaction with the environment is consciousness and intentionality, which are linked to values, goals, and emotions – and the fact that the individual learns from experience. These circumstances must be taken into account when methods derived from the study of dynamic, complex processes in the physical world are applied to the planning and implementation of empirical research on developmental processes. (Magnusson, 2001, p. 161)

Along partly similar lines, Deacon (2012) makes a differentiation between *morphodynamic* and *teleodynamic* systems. To illustrate: Living organisms are teleodynamic systems, whereas the weather is a morphodynamic system. Teleodynamic systems such as living organisms are *organized with respect to a vast world of possible future events and abstract properties*, whereas the weather “is organized only with respect to the immediate physical conditions” (p. 41). The organizing principle of teleodynamics also means that the individual organism is organized to maintain and perpetuate itself and this very organization. Deacon speaks about this as “reflexive individuation”; only living organisms are reflexively individuated in the sense that their processes are organized in the service of their own self-maintenance.

A basic characteristic of what Deacon (2012) refers to as teleodynamic systems is that they are organized with respect to a world of *possibilities*. This is consistent with what can be seen at all levels of psychological functioning, from perception and thinking to life plans. Possibilities enter the scene already in our perception of the environment. In Gibson’s (1979) ecological approach to perception the most important information that is provided via our senses is information about the environment’s *affordances*, defined as what the environment affords the individual in terms of *possible* interactions, for good or bad. Affordances are what the environment “offers the animal, what it provides or furnishes, either for good or ill” (Gibson, 1979, p 127). Some simple examples are what the environment offers in terms of *possible action*: an open environment affords locomotion in any direction over the ground; objects of a certain shape and size afford sitting; different kinds of tools afford construction, manipulation, etc.

Another example can be taken from social psychology. Markus and Nurius (1986) introduced the concept of *possible selves* as a name for the individual person’s ideas of what they *might* become, what they *would like* to become, and what they are *afraid of* becoming, and that may thereby function as incentives for future behavior. Concepts such as purposes, ambitions, ideals, and many others, refer to future possibilities and point the way to some essential characteristics of persons.

Human beings’ ability to *reflect about possibilities* is also central to all kinds of scientific endeavours, from mathematics and logics to empirical sciences and phenomenological reflection. In the empirical sciences this is seen, for example, in scientists’ ability to formulate various kinds of hypotheses and deduce implications from these hypotheses that can be tested by empirical means. All kinds of theoretical analyses involve a reasoning about possibilities and the logical implications of these.

At a less scientific level, human elaboration on *possible* lives and life experiences abounds in fiction literature. This is sometimes described as a rich source of psychological insights. The historian Lynn Hunt (2007) has suggested that the reading of fiction literature may be one way of training the ability to empathize with other people’s experiences, including those from different backgrounds and different cultures that we do not ordinarily have access to. Among other things, Hunt raises the question if it was merely a coincidence that the breakthrough of the novel during the 18th century was followed by an increased public discussion of human rights and an increased critique of the use of torture.

It should also be noted that, although person-level research is much underrepresented in psychological science, there is a large psychological literature about issues at the person level, in the form of books (rather than scientific papers) written by clinical psychologists, psychotherapists and psychoanalysts. As a result, the field of clinical psychology contains a wild flora of theories about personal development and interpersonal processes. Many of these theories have been developed “intuitively” by clinicians based on their experiences with patients, whereas others have been developed through phenomenological reflection by philosophically minded theorists. These theories are only to a very limited extent tested in empirical research.

This situation is sometimes described as there being a *gap* between empirical research and clinical practice (e.g., Teachman et al., 2012). Although the person-level is little represented in empirical psychological research, it is quite central to clinical psychological practice, because clinical psychologists need theories about personal development and interpersonal relations that can guide them in their work. To the extent that they cannot find such guidance in academic psychological research they base their practice on theories that have their origin elsewhere. It is essential, however, that clinical psychological practice rests on well-founded theories that make it possible to understand individual persons and their experiences, their interpersonal relations, and their interaction with the environment as validly and efficiently as possible. This is a main reason why more research needs to be focused on the person level.

To summarize: If the research questions at the population level often are about the prevalence of various psychological phenomena, and the research questions at the mechanism level ask for explanations of various aspects of psychological functioning, a main research question at the person level is how we can develop *valid models for analyzing* individual persons, interpersonal relations, and other aspects of person-environment interaction (see Table 1).

**Table 1:** Levels of psychological research, as exemplified by research questions at each level.

Levels of psychological research	Examples of research questions
Population psychology	How common is…? (e.g., mental disorders, attitudes, values) How has prevalence changed over time, and why? How effective are psychological treatments (e.g., in terms of percentages of patients who respond to the treatment, and compared to control conditions)?
Person psychology	How to describe and analyze the person as an intentional agent in interaction with the environment, as part of interpersonal relations, and in development and change? How to describe and analyze the kind of interaction between therapist and patient that leads to change in psychotherapy?
Mechanistic psychology (sub-personal level)	How to explain psychological functioning (e.g., perception, memory, learning, emotion, psychological development) in terms of underlying (neurophysiological or information processing) mechanisms? How to explain psychological change in psychotherapy in terms of changes in internal mechanisms (in the brain, or in information processing terms)?

## Conclusion

A general conclusion from the preceding discussion is that, although population psychology and mechanism psychology are well-established research areas with clear research question and methodologies, this is not equally true of person-level psychology. Related to this, there is tendency among some researchers to search for answers that belong to the person-level either at the population level or the mechanism level. This failure to differentiate clearly between research questions belonging to the three levels lead to questionable research practices.

Most notably, a failure to differentiate clearly between the population level and the person level leads to problem-method mismatches so that researchers try to answer questions about persons by means of research on populations. Also, because of a failure to differentiate between the person level and the mechanism level, explanations in terms of subpersonal mechanisms are too often assumed to be sufficient to understand processes at the person level. A clear differentiation between three levels of psychological science – population psychology, person psychology, and mechanism psychology – in terms of the research questions involved and may contribute to an increased clarity in these matters. Most importantly, much more research needs to be focused on the person level.

## Action editor

Lars R. Bergman served as action editor for this article.

## Open access

This article is distributed under the terms of the Creative Commons Attribution 4.0 International License (http://creativecommons.org/licenses/by/4.0/), which permits unrestricted use, distribution, and reproduction in any medium, provided you give appropriate credit to the original author(s) and the source, provide a link to the Creative Commons license, and indicate if changes were made.
